# Diaphragmatic Peritonectomy versus Full Thickness Diaphragmatic Resection and Pleurectomy during Cytoreduction in Patients with Ovarian Cancer

**DOI:** 10.1155/2013/876150

**Published:** 2013-12-18

**Authors:** P. N. J. Pathiraja, R. Garruto-Campanile, R. Tozzi

**Affiliations:** Department of Gynaecologic Oncology, Oxford Cancer Centre, Churchill Hospital, Oxford University Hospital, Oxford OX3 7LJ, UK

## Abstract

*Objectives*. Compare the surgical morbidity of diaphragmatic peritonectomy versus full thickness diaphragmatic resection with pleurectomy at radical debulking. *Design*. Prospective cohort study at the Oxford University Hospital. *Methods*. All debulking with diaphragmatic peritonectomy and/or full thickness resection with pleurectomy in the period from April 2009 to March 2012 were part of the study. Analysis is focused on the intra- and postoperative morbidity. *Results*. 42 patients were eligible for the study, 21 underwent diaphragmatic peritonectomy (DP, group 1) and 21 diaphragmatic full thickness resection (DR, group 2). Forty patients out of 42 (93%) had complete tumour resection with no residual disease. Histology confirmed the presence of cancer in diaphragmatic peritoneum of 19 patients out of 21 in group 1 and all 21 patients of group 2. Overall complications rate was 19% in group 1 versus 33% in group 2. Pleural effusion rate was 9.5% versus 14.5% and pneumothorax rate was 14.5% only in group 2. Two patients in each group required postoperative chest drains (9.5%). *Conclusions*. Diaphragmatic surgery is an effective methods to treat carcinomatosis of the diaphragm. Patients in the pleurectomy group experienced pneumothorax and a higher rate of pleural effusion, but none had long-term morbidity or additional surgical interventions.

## 1. Introduction

Ovarian cancer remains a lethal disease for patients with advanced disease. Despite medical progresses, the survival figures of ovarian cancer did not significantly improve [1]. Whilst every year 200.000 women are diagnosed with ovarian cancer globally, 125.000 will die of disease [2]. The lack of an effective screening test and a delayed diagnosis are the reasons for this high rate of lethality. In fact, over 75% of the patients will be allocated to a FIGO stage III or IV at time of diagnosis, with involvement of the upper abdomen. The residual disease following surgery remains the single most important independent prognostic factor in patients with ovarian cancer [3–6], regardless of the timing of the surgery [7]. The lesser the residual disease at the end of the surgery, the better is the prognosis. Patients left with no visible disease are associated with the best outcome [5–9]. In order to achieve a complete extirpation of the cancer, often a multivisceral surgery is necessary [10]. Despite being anatomically distant from the organ of origin, the upper abdomen and the diaphragm are often involved in patients with ovarian cancer. A survey of the Society of Gynaecologic Oncology based on 1965 patients with ovarian cancer indicated the presence of diaphragmatic disease to preclude an optimal cytoreductive surgery in 76% of the cases [11]. Traditionally, upper abdominal surgery is not included in the surgical portfolio of gynaecologists. Therefore, diaphragmatic disease and pleural involvement can potentially leave many patients with suboptimal cytoreduction despite complete clearance of the pelvis [11–13]. With more evidences in support of a surgical effort aimed at no residual disease, the expertise for upper abdominal and diaphragmatic surgery has become important [14]. Several studies have reported on the resection of diaphragmatic disease [9]. Based on the extension of the disease, surgery of the diaphragm can be limited to a peritonectomy or require a full thickness resection of the muscle and the pleura. In this study we compare the surgical morbidity of patients underwent diaphragmatic peritonectomy with that of patients underwent a full-thickness resection of the diaphragm and pleura during multivisceral cytoreduction for ovarian cancer.

## 2. Patients and Methods

The study obtained the approval of the Trust Clinical Governance Department. We use the hospital surgical database to prospectively record all the surgical data. The study group includes all patients with FIGO stage IIIC/IV invasive ovarian, fallopian tube, and peritoneal carcinoma patients undergone diaphragmatic surgery during multivisceral cytoreduction at our institution between April 2009 and February 2012. Preoperative exclusion criteria for multivisceral cytoreduction were an American Society of Anesthesiology (ASA) risk assessment score >3, a performance status >2, and metastases in the lungs and/or in 3 or more liver segments as per CT review at the Gynaecologic Oncology multidisciplinary meeting. As an additional triage method, all patients underwent an explorative laparoscopy just before the laparotomy. The aim was to identify patients with disease on 2 or more metres of small bowel serosa or encasing the porta hepatis precluding optimal debulking. All the patients in this series were identified from the surgical database having had either diaphragmatic peritonectomy, full thickness resection of diaphragm and pleura, or both. Based on the type of surgery, the patients were divided into group 1 (diaphragmatic peritoneal stripping—DS) and group 2 (diaphragmatic full thickness resection with pleurectomy—DR). We do not perform coagulation of disease using diathermy or any other mode; this is because we believe excision by surgery may be superior to other destructive methods. Patients who underwent debulking procedures without diaphragmatic surgery or those who had coagulation to peritoneal disease were excluded from the study. Patients who were not suitable for radical debulking in primary setting or after chemotherapy were referred to the medical oncology team immediately to continue with medical treatment.

The endpoint of this study was the surgical morbidity. Hence all patients assess undergone diaphragmatic surgery were included regardless of the timing (primary or after chemotherapy) of the surgery. A complete or optimal cytoreduction was defined as no visible tumor (R0). The size of residual tumor if any was visible had to be recorded in the frame of a suboptimal cytoreduction. The surgical technique of multivisceral cytoreduction is fully described elsewhere. In summary, the procedure, performed by means of a xifopubic laparotomy, aims at cleaning the abdomen of any visible tumour. Starting from the pelvis, any additional procedure is dictated by the presence of visible tumour. The diaphragmatic surgery will be described in more detail. An initial inspection is done by gentle retraction of the right liver. However, in most cases the liver mobilization is necessary to assess the real extent of the diaphragmatic disease. We use Bookwalter's retractor for all radical debulking procedures to have good surgical exposures and to free the assistants for surgery. The procedure involves resection of the falciform, right coronary and triangular ligaments of the liver. The decision on the starting point, that is, the supra- or infrahepatic portion of the liver attachments, is based on where the least amount of disease is present. The aim of the liver mobilization is to fully expose the diaphragmatic disease and, most importantly, to access the bare area of the liver. In some patients this mobilization is uncomplicated and sufficient to perform the diaphragmatic surgery. Often, however, the disease is adherent or inseparable from the Glisson capsule. In this case or in patients where the diaphragmatic disease is very close to the vascular structures of the liver, a complete vascular control is advisable. In these patients, we do not attempt to separate the diaphragmatic disease from the liver capsule, as it will generate a diffuse bleeding and hide the right surgical plane. A formal dissection of the liver hilum is then performed with the Pringle maneuver and a vessel loop is positioned to isolate the hepatic hilum. Clamping of the hilum, if necessary, is applied for 15 continuous minutes with a release time of 5 minutes. In addition, the infrahepatic portion of the IVC is identified and followed to dissect the hepatocaval ligament. Usually the vascular structures are not involved by the tumor as they remain retroperitoneal. The dissection continues along the superior aspect of the IVC until the posterior wall of the right hepatic vein is found. At this stage the right hepatic vein is identified and, if involved by the disease, it is encircled with a vessel loop. The middle and left hepatic veins are rarely involved and it is usually sufficient to visualize them. Finally the diaphragmatic surgery begins with monopolar incision of the diaphragmatic peritoneum well away from the disease. The dissection is carried out moving towards the center with traction over clamps and bipolar coagulation along the plane between the peritoneum and the muscle. Usually the upper pole of the right kidney, the adrenal, and the right wall of the IVC are exposed. When the disease is inseparable from the Glisson capsule, then the entire diaphragmatic peritoneum is mobilized until it is fully detached from the muscle. Once the vascular control is obtained as previously described, the dissection plane is sought between the vessels and the disease. At this stage with the vessels fully exposed, the traction is applied moving away from the centre. Having created a “bag of peritoneum,” only then the disease is dissected from the mobilized liver. In some patients, it is sufficient to remove the Glisson capsule. In others, a nonanatomic hepatic resection is required. We usually avoid stripping off the liver tissue as it would end in diffuse bleeding. The decision to perform a diaphragmatic full thickness resection is most commonly taken during the surgery. It is based on the finding of a full thickness invasion of the muscle by the tumor or tenaciously adherent to the muscle precluding the peritoneal stripping. The full thickness resection is performed by accessing the pleural cavity with bipolar scissor. A full inspection of the pleural cavity follows to explore for additional disease of the pleura. During this phase, to allow for a good exposure of the pleural cavity, the respiratory rate is maintained at around 10 acts/min. The pleural resection has to include all the visible disease aiming for free margins regardless of the size of the resection. The pleural cavity is closed with a continuous 0 PDS full thickness suture. Before closing, a 10 Foley catheter is placed in the pleural cavity and the anesthetist starts a manual ventilation to provide maximal expansion of the lungs (Valsalva maneuver). The aim is to recreate the negative pressure by suction through the Foley catheter and by maximal expansion of the lungs. The ultimate goal is to avoid a pneumothorax. The suture is tied as a purse string around the Foley catheter which is removed at the very end. The integrity of the suture line is checked with an air test by filling the space with water and requiring a last Valsalva maneuver. During this procedure we would keep the patient tilted to the right with minimal Trendelenburg.

We used the Clavien-Dindo classification for surgical morbidity [15] defining intraoperative complications as adverse events occurring within 30 days from the surgery. Pneumothorax and pleural effusion are the most common complications of diaphragmatic surgery [16–24]. Hence the study protocol screened all patients with serial chest radiography starting on the first postoperative day and repeating later on if the initial was positive. The presence of a pleural effusion was defined as the presence of at least 100 cc of fluid in the pleural cavity. An obliteration of the costophrenic space was not considered as pleural effusion. The data were analyzed using the chi-square test or Fisher's exact test for categorical variables and the Student's *t*-test for continuous variables. A *P* value of 0.05 or less was considered statistically significant.

## 3. Results

The total number of patients included in the study is 42, equally divided into groups 1 and 2.  Table 1 shows the patients and the tumor characteristics. There were no significant differences in the 2 groups. There was a total of 117 advanced stage ovarian cancer patients who had surgical assessment for debulking and five patients did not proceed to full debulking procedure after laparoscopic assessment due to wide-spread small bowel serosal involvement (5/117). Only those who had diaphragmatic peritonectomy were included in the study: 42 out of 112 patients (37.5%).

Table 2 shows the surgical outcomes and hospital stay. The extent of surgery was similar in both groups. For example, bowel resections were carried out in 11 out of 21 patients in group 1 and 18 out of 21 in group 2, *P* = 0.62. The median duration of the entire multivisceral cytoreduction was 387 versus 451 minutes, median blood loss 731 mL (range 200–3000) versus 747 mL (range 300–1500), and hospital stay 14 days (range 4–45 days) versus 12 days (range 6–32 days). None of the differences was statistically significant. Complete tumour resection (*R* = 0) was achieved in 19 out of 21 patients (90.4%) in group 1 and in 20 out of 21 patients (95.2%) in group 2, *P* = 0.93. In all 3 patients with residual disease, it never exceeded 5 mm. Four patients in each group needed a nonanatomic or wedge liver resection to achieve a complete tumor resection. None of the patients had chest drain placed intraoperatively. Five patients out of 42 (11.9%) developed a pleural effusion only detected on the routine chest X-ray. Two patients were in group 1 and 3 in group 2, *P* = 0.87. None of the five patients was symptomatic and all were managed conservatively. Final histopathology confirmed the presence of cancer on the peritoneal surface of 19 out of 21 patients in group 1, whilst no cancer was found in 2 out of 21 patients who were operated on after 3 cycles of chemotherapy. In group 2, all 21 patients had peritoneal involvement, with 11 out of 21 (52.3%) also having cancer on the pleural surface. One patient per group suffered from a breakdown of the bowel anastomosis. Wound dehiscence up to the rectus sheath occurred in two patients of group 1 and three patients of group 2. Other 2 patients had rare but severe complications that are worth mentioning. One patient in group 1 developed a postoperative ureteric fistula, which was not diagnosed until day 9 due to a very low output. The patient eventually developed urosepsis and possibly succumbed due to the complications of the fistula. This patient underwent, during the cytoreductive surgery, the resection of a 6 cm retroaortic lymph node encroaching on the aorta, the bifurcation of the common iliac vein, and grossly distorting the course and the caliber of the left ureter with severe hydronephrosis. In group 2 one patient had a dehiscence of the pleural suture on day 3 following pleurectomy during the cytoreductive procedure. The radiological findings suggested the herniation of the liver in the thorax. A second laparotomy and a thoracotomy with a full repair were required. After a complex postoperative time with 10 days spent in intensive care, she recovered well. At time of second surgery, a thin suture material was found other than the usual 0 PDS. This was thought to be the possible cause of the dehiscence, otherwise performed with the standard technique. The presence of a pneumothorax was detected in 3 out of 42 patients (7.1%) and it was only seen in group 2. The largest area of pneumothorax was 2 cm and all patients were managed conservatively except the patient with the suture dehiscence. Both groups suffered one mortality, during the postoperative period: one of them due to sepsis as described and the other suffered an anastomotic leakage, sepsis, and multiorgan failure. Grade of compilations and types of complications are listed in Tables 3 and 4.

## 4. Discussion

The residual disease after the surgery remains the most powerful prognostic factor of survival in patients with ovarian cancer. The results of a clinical trial conducted by the EORTC have confirmed the value of the surgical effort in both study arms, after neoadjuvant chemotherapy or primary surgery [7]. Before the publication of these prospective data, several retrospective studies emphasized the prognostic value of a complete cytoreduction [3–14, 25]. The surgical targets of residual disease decreased over the years from <2 cm to 0.5 cm. Lately a few studies have proved the benefit of the surgical resection to be most striking in patients left with no visible disease [4–6]. In the frame of a complete resection, surgery of the upper abdomen is inevitable. In fact over 70% of the patients with ovarian cancer have upper abdominal disease at time of diagnosis. Some retrospective studies provided evidence of a survival benefit when upper abdominal surgery and diaphragmatic resection were added to achieve complete resection [10, 16, 26].

During the study period there was change of practice from primary cytoreductive surgery to interval cytoreduction after 3 cycles of chemotherapy; this might explain the reduced number of diaphragmatic peritonectomy (37.5%) in this patient series.

Both the diaphragmatic peritonectomy and/or resection with pleurectomy have been previously reported [16–19, 26]. In the present study we met a relatively low complication rate and only few events were directly caused by the diaphragmatic surgery. Previous studies suggested postoperative pleural effusions and pneumothorax to be the most common complications of diaphragmatic surgery [17–23]. Within the published papers, the rate of patients with pleural effusion is extremely variable, ranging from 10% to 59% [17–23]. However, some of the studies protocol included the elective placement of chest tubes in case of large diaphragmatic resections. This most likely reduced the occurrence of a pleural effusion as the pleura was drained. In our study, the protocol did not include a chest drain regardless of the resection size. Despite large resections, only one patient out of 21 (4%) developed pleural effusion needing surgical intervention with chest drain. Previous studies indicated the following as prognostic factors for postoperative pleural effusion: opening of the pleura [14] and liver mobilization [20]. In our study none of the two was a significant predictor of pleural effusion: all patients had liver mobilization and patients in group 2 did not have a higher rate of pleural effusion. Final histology revealed that 11 patients out of 21 (52.3%) had tumour involvement of the pleural surface. Only 2 out of 10 patients with negative pleural specimen had primary surgery, while 8 out of 10 had surgery following chemotherapy. We drew two conclusions from these results. One is that diaphragmatic peritonectomy alone would leave disease behind, that is, on the pleural surface, in 26% of the patients had a pleural resection not been performed. The second illustrates the difficulty in the intraoperative assessment of the extension of the disease. At times full thickness resection is inevitable due to the dense adhesion of the tumour to the diaphragmatic muscle or pleura. This remains particularly true for patients operated on following chemotherapy. The decision to perform a pleural resection in patients operated on after chemotherapy is challenging and not always supported by the presence of disease on the pleural surface. Despite a low rate of surgical complications, the risk associated with the breakdown of a diaphragmatic repair, a potentially life threatening complication, is worth emphasizing. Such complication has not been reported so far in patients with ovarian cancer. In our patient it may have occurred due to inappropriate suture material used during the closure.

## 5. Main Findings and Limitations

The aim of this study was to investigate the morbidity associated with diaphragmatic peritonectomy and/or full thickness resection of the pleura. We found no statistically significant difference between intra- and postoperative complications. Diaphragmatic surgery contributed to achieve optimal debulking in about 95% of the patients despite the presence of widespread disease. Despite the prospective nature of the study and the effort to avoid a selection bias, the lack of randomization may limit the validity of the findings.

## 6. Conclusion

Diaphragmatic peritonectomies and full thickness resections of the diaphragm with pleurectomy are effective methods to treat carcinomatosis of the diaphragm. The patients in the pleurectomy group experienced pneumothorax and a higher rate of pleural effusion, but none had long-term morbidity. *Practice Points*. The use of peritonectomy or full thickness resection with pleurectomy has to be modulated by the type of disease. However, both procedures are often required and the expertise should be available when attempting cytoreductive surgery with the aim of no residual disease. *Research Recommendations*. (i) Monitoring the outcomes of diaphragmatic surgery may be important as these procedures become more frequent in debulking surgery. (ii) Improve the diagnostic power in the detection of pleural disease.

## Figures and Tables

**Table 1:** Patients and tumor characteristics.

	Group 1 (DS) *n* = 21	Group 2 (DR) *n* = 21
Age mean, years	64	63.5
FIGO stage		
IIIc	20	14
IV	1	7
Tumour histology		
Serous high grade	19	15
Clear cell	1	2
Adeno. Ca	1	0
Endometrioid	0	3
MMT	0	1
CA 125	1339	572

**Table 2:** Surgical outcomes and hospital stay.

Surgical data	Group 1 (DS) *n* = 21	Group 2 (DR) *n* = 21
Surgical time (minutes)	387	451
R0 (patients)	19	20
R1 (patients)	2	1
Blood loss (cc)	731	743
Large bowel (patients)	11	18
Small bowel	2	3
Anastomosis	9	16
Bowel diversion	3	3
Permanent stoma	1	2
Liver resection	4	4
Cholecystectomy	0	1
Splenectomy	1	4
Hospital stay	13	12

**Table 3:** Complications according to the Clavian-Dindo classification.

Grade	Group 1	Group 2
Grade 1-2	2	1
Grade 3a	1	3
Grade 3b	0	1
Grade 4a	0	1
Grade 4b	0	0
Grade 5	1	1

Total	19%	33%

**Table 4:** Complications according to groups.

Type of complication	Group 1	Group 2
Wound dehiscence	2	3
Anastomotic leak	1	2
Ureteric fistula	0	1
Pleural effusion	1	1
